# Spatial organization and nuclear positioning of murine immunoglobulin loci in developing B cells

**DOI:** 10.1186/1756-8935-6-S1-P71

**Published:** 2013-03-18

**Authors:** Magdalena B Rother, Kevin AM van Kester, Jacgues JM van Dongen, Cornells Murre, Menno C van Zelm

**Affiliations:** 1 Department of Immunology, Erasmus MC, University Medical Center Rotterdam, the Netherlands; 2 Division of Biological Sciences, Department of Molecular Biology, University of California, San Diego, La Jolla, CA 92093, USA

## Background

Genomic DNA in eukaryotic cells is highly organized and shows multiple levels of compaction. Contraction and folding of DNA enables long-range interactions between widely dispersed genes to facilitate their expression. Furthermore, genes are positioned in the nucleus to either transcriptionally active or permissive compartments [1]. During B-cell development in the bone marrow, immunoglobulins (Ig) are assembled through stepwise recombination of V, (D) and J genes. Imaging studies have shown that Ig loci are organized into rosette-like clusters of loops and contract prior to rearrangement [2]. Moreover, during B cell development Ig loci change their nuclear positioning [3]. However, how the chromatin fiber is organized into higher-order structures and how this is regulated is still unknown.

## Material and methods

To study 3D organization and nuclear compart-mentalization of Ig loci, we performed 3D DNA FISH on progenitor B cells derived from E2A-/-, RAG1-/- and RAG1-/-Vh81X mice. Three regions along the *IGH* and *IGK* loci were detected with BAC probes. Spatial distances between genetic markers and from each probe to the nuclear lamins were measured with a confocal microscopy and analyzed.

## Results

Spatial distant measurements between *IGH* and *IGK* probes showed that both loci were contracted in RAG1-/- pro-B cells as compared with E2A-/- pre-pro-B cells. Interestingly, both loci remained contracted in RAG1-/-Vh81X pre-B cells. The nuclear gene positioning analysis revealed that *IGH* moved from the nuclear periphery to the central domains in pro-B cells, while *IGK* remained peripherally located. Similarly, *IGK* relocated in pre-B cells to nuclear center, whereas *IGH* repositioned peripherally. However, in other cell stages both loci were kept in the peripheral compartments.

## Conclusions

Our studies confirm that spatial chromatin organization of murine *IGH* and *IGK changes* during development. Both loci undergo contraction prior to gene rearrangement which allows juxtaposing of genomically distant gene segments and creates equal opportunities for recombination. Specifically, *IGH* and *IGK* were contracted in pro-B cells, although *IGK* is assembled later during the development. This observation can be explained by changes in the nuclear gene positioning. *IGH* and *IGK* were tightly associated with the nuclear lamins in early pre-pro-B cells. However, in committed pro-B and pre-B cells, *IGH* and *IGK* were consecutively positioned to the active central compartments. Hence, we conclude that spatial chromatin organization and nuclear positioning orchestrate stepwise antigen receptor formation. Chromosome conformation capture experiments are ongoing to further dissect the DNA folding mechanism.
